# Anesthetic management of fetal pulmonary valvuloplasty: A case report

**DOI:** 10.1515/med-2023-0835

**Published:** 2023-11-02

**Authors:** Xiaofeng Lei, Xuezhu Huang

**Affiliations:** Department of Anesthesiology, Women and Children’s Hospital of Chongqing Medical University, Chongqing Health Center for Women and Children, Chongqing, China

**Keywords:** pulmonary valve stenosis, anesthesia for fetal surgery, combined spinal epidural anesthesia, fetal surgery, intrauterine surgery

## Abstract

Anesthesia management of fetal pulmonary valvuloplasty (FPV) is difficult, requiring careful consideration of both the mother and the fetus. Few reports have been published on specific anesthesia implementation and intraoperative management. We report the case of a pregnant woman who was treated with FPV under combined spinal epidural anesthesia (CSEA) with dexmedetomidine in the second trimester of pregnancy. Meanwhile, the application of fetal anesthesia through the umbilical vein was optimal. During the operation, the vital signs of the pregnant woman were stable with no complications and the fetal bradycardia was corrected by intracardiac injection of epinephrine. Four months postoperatively, a boy was born alive by full-term transvaginal delivery. CSEA may be a suitable anesthesia method for FPV surgery. Nevertheless, maternal hemodynamic stability maintenance, effective fetal anesthesia, and timely fetal resuscitation were necessary.

## 1 Introduction

Fetal and neonatal pulmonary atresia with intact ventricular septum (IVS) or critical pulmonary stenosis (CPS) is characterized by high mortality. Fetal pulmonary valvuloplasty (FPV) can promote the right heart development, reduce postnatal mortality, and improve long-term prognosis [1]. Anesthesia for FPV is challenging as it is to be realized simultaneously in pregnant women and fetuses. Anesthetic care needs to take into account not only maternal and fetal physiology but also the anesthetic drugs’ interaction with the maternal–fetal health [2].

Thus, the safety of pregnant women and the provision of painless surgical conditions are to be ensured. Besides, to facilitate the operation, the fetus should not be able to exert movement, and the adverse stress reaction and pain caused by the puncture needle piercing the fetus should be reduced [3]. Reports on the anesthetic management of FPV mothers and fetuses are scarce or the anesthesia procedure was not detailed enough [4]. In this case, an FPV under combined spinal epidural anesthesia (CSEA) in the second trimester is reported.

## 2 Case presentation

A 29-year-old pregnant woman with G2P0 was admitted to the Women and Children’s Hospital of Chongqing Medical University (Chongqing, China) in June 2022 with CPS/IVS at 25 + 2 weeks of gestation. FPV under ultrasound guidance was considered. The mother and the multidisciplinary team reached an agreement that once the situation was dangerous for pregnancy due to surgical complications, cesarean section and resuscitation of the preterm infant would be performed in time. When we discussed the anesthesia methods with the mother, she firmly refused the general anesthesia for psychological causes: her friend died for complications of general anesthesia. After entering the operating room, pure oxygen (5 L/min) was inhaled through a mask.

The left radial artery was punctured and catheterized for invasive arterial monitoring. CSEA was performed at the L3–4 intervertebral space: 2 mL of 0.5% ropivacaine was injected into the subarachnoid space (2 mL of 0.75% ropivacaine + 1 mL of self-cerebrospinal fluid). Then, 4 cm epidural catheter was inserted into the cephalad epidural space. The position was adjusted to ensure that the plane of anesthesia was fixed at T8. Intravenous infusion of dexmedetomidine was performed for sedation (a loading dose of 0.5 µg/kg for 10 min and a maintenance dose of 0.3 µg/kg/min). The fluctuation range of invasive arterial blood pressure was within 20% of the basal blood pressure (131/77 mmHg). The heart rate was 73–100 beats/min. The amount of fluid intake was limited appropriately: a total of 300 mL of crystalloid solution was infused during the operation. The operation lasted 40 min. During the operation, the pregnant woman was emotionally stable, quiet, and experienced no discomfort. Two hours after the operation, her muscle strength and sensation of the lower limbs were fully recovered, with no complications such as urinary retention or nerve damage. The Visual Analogue Scale postoperative scores for pain were all less than 3.

Fetal anesthesia was performed under ultrasound-guided umbilical vein injection of 10 µg of fentanyl and 0.1 mg of vecuronium bromide. The ultrasound system monitored fetal heart rate (FHR) continually. The fetal position was well fixed during the operation, with no body movement affecting the operation. When the balloon catheter was dilating the pulmonary valve, the FHR slowed down to 92 beats/min. Meanwhile, the maternal vital signs remained stable. In consideration of that the fetal bradycardia was caused by dilating, the procedure was stopped immediately. But the FHR was still less than 100 beats/min within seconds. After an immediate intracardiac injection of 1 µg of epinephrine through the balloon catheter, the FHR returned to normal level. Four months postoperatively, a boy was born alive by full-term transvaginal delivery.


**Informed consent:** Informed consent has been obtained from all individuals included in this study.

## 3 Discussion

With the development of medical technology and the improvement of social conditions, the numbers of clinical interventions for fetal surgery have been increasing. Currently, controversy exists concerning the anesthesia method to be applied during FPV [1, 5–7]. Maternal general anesthesia without separate fetal analgesia has been reported successfully in FPV [1], but maternal–fetal hemodynamic instability and fetal myocardial depression caused by high-dose opioids and inhaled anesthetics in general anesthesia should be considered [8,9]. There were no prospective studies to compare the general anesthesia and the intraspinal anesthesia in FPV. In addition, the patient’s refusal made the general anesthesia hard to be performed in this case.

Epidural anesthesia (EA) was associated with stable hemodynamic conditions and appeared to be a suitable technique for fetoscopy such as aortic valve angioplasty [5], but the single EA was not the preferred anesthetic technique in this case, because the mother expressed her willingness that once the situation was dangerous for pregnancy, cesarean section and resuscitation of the preterm infant would be performed in time. It meant that the urgent cesarean section might be performed at any time during surgery, and muscle relaxation was important in this procedure, while the spinal anesthesia could provide better analgesia and muscle relaxation than EA [10,11]. Moreover, CSEA was a mature and applicable technique in our institution, and previous studies have demonstrated that subarachnoid injection of ropivacaine at the low dose was used to avoid the intensive hemodynamic changes caused by spinal anesthesia and the stable hemodynamic effect was obtained in pregnant women [12,13].

Considering we were not experienced for this invasive fetal surgery and the length of operating was uncertain, local anesthetic infiltration was not the appropriate option as it failed to satisfy long-time surgery or provide adequate analgesia and muscle relaxation. The small-dose CSEA was safe and effective in our case and could provide better muscle relaxation when emergency cesarean section was necessary during FPV, and long-time EA could be achieved by the reserved epidural catheter.

The gestational age at which the fetus perceives pain is controversial. Nevertheless, noxious stimuli at 18–20 weeks of gestational age induced stress responses and hemodynamic changes, which led to fetal bradycardia and compensatory redistribution of the blood flow from the peripheral tissues to the brain, heart, and placenta [14]. In addition, fetal stress response increased uterine sensitivity and led to preterm birth [3]. Therefore, fetal anesthesia is necessary in fetal invasive surgery, which can reduce not only pain but also the noxious stress response. In this case, “cocktail therapy,” a combination of fentanyl and vecuronium bromide, was used for fetal anesthesia [15]. No movement of the fetus was observed during the whole procedure. The pregnancy was stable and the fetus developed well during the postoperative follow-up period.

The incidence of cardiac depression during fetal surgery is high [9]. In this study, the Doppler ultrasound system was used to closely monitor the FHR. Fetal bradycardia was the main fetal complication. Preoperative preparation for fetal resuscitation was required. Compared with intramuscular and umbilical vein injections, intracardiac injection of epinephrine was decisively used for resuscitation, achieving a faster and more pronounced positive effect [3].

Normal maternal hemodynamic parameters in FPV are critical for the maintenance of adequate uteroplacental blood flow [16]. In this case, ropivacaine at a low dose was injected in the subarachnoid space to reduce the supine hypotension syndrome or the intensive hemodynamic changes, and afterwards, the stable hemodynamic parameters were achieved: blood pressure fluctuation range of the pregnant woman was within 20% of the baseline and that the heart rate was 73–100 beats/min. Besides, an invasive arterial channel was established preoperatively to continuously monitor arterial blood pressure. Compared with noninvasive blood pressure, invasive blood pressure was more sensitive and rapid in response to hemodynamic changes and this advantage could lead to more timely correction of maternal hypotension which might cause low uteroplacental blood flow.

The pregnant woman who underwent FPV in the present investigation complained of severe preoperative anxiety and stress. Therefore, we choose intraspinal anesthesia with auxiliary sedation to relieve her tension. We achieved a good sedative effect by the use of dexmedetomidine intravenous infusion. The blood pressure and heart rate of the pregnant woman remained stable, with no complications or discomfort during the anesthesia process.

In conclusion, low-dose CSEA may be considered an appropriate technique for FPV minimally invasive surgeries. Nevertheless, it is critically important to implement effective fetal anesthesia and prompt fetal resuscitation. Maternal hemodynamic stability should be maintained throughout the operation. Dexmedetomidine can achieve ideal sedation in pregnant women treated with FPV under CSEA.
